# Correction: 12-year observation of tweets about rubella in Japan: A retrospective infodemiology study

**DOI:** 10.1371/journal.pone.0294796

**Published:** 2023-11-16

**Authors:** Yukie Sano, Ai Hori

There are errors in the Funding section. The correct Funding statement is: Y.S. received the Japan Society for the Promotion of Science KAKENHI Grant Number 20K19928 and 23H03502. A.H. received the Japan Society for the Promotion of Science KAKENHI Grant Number 20K10467 and Health Labour Sciences Research Grant number 21HA2014. The funders had no role in study design, data collection and analysis, decision to publish, or preparation of the manuscript.
