# Supplementary material for: 12-year observation of tweets about rubella in Japan: A retrospective infodemiology study
Source: PLoS One. 2023 May 8;18(5):e0285101. doi: 10.1371/journal.pone.0285101 (PMC10166556; doi:10.1371/journal.pone.0285101)
Supplement: Attachment — Submitted filename: PONE-D-22-33446_reviewer R.pdf [file pone.0285101.s002.pdf]

PLOS ONE

Long-term observation of social media posts about rubella in Japan

--Manuscript Draft--

**Manuscript Number:**	PONE-D-22-33446
**Article Type:**	Research Article
**Full Title:**	Long-term observation of social media posts about rubella in Japan
**Short Title:**	Long-term observation of social media posts about rubella in Japan
**Corresponding Author:**	Yukie Sano<br>Tsukuba Daigaku - Tsukuba Campus: Tsukuba Daigaku<br>Tsukuba, Ibaraki JAPAN
**Keywords:**	social media; Twitter; Public Health; rubella
**Abstract:**	Although rubella is an infectious disease that can be prevented by vaccination, there have been periodic epidemics in Japan, mainly among adult males. One of the reasons for this is the lack of interest in vaccination among the target adult male population. To empirically observe interest in rubella, we collected and analyzed Twitter posts about rubella in Japanese between January 2010 and May 2022. We found that the weekly number of rubella reports and the number of Twitter posts fluctuate simultaneously, with an increase in both during the 2012-2013 rubella epidemic. Furthermore, during the 2018 rubella epidemic, the number of tweets increased due to the start of the rubella routine vaccination program and the use of cartoons to raise awareness. While 80% of the accounts posted three times or fewer during the period, some accounts posted multiple times per day for more than 12 years. Most of the content posted was medical information, on topics such as vaccines and antibodies.
**Order of Authors:**	Yukie Sano
	Ai Hori
**Additional Information:**	
**Question**	**Response**
**Financial Disclosure**<br><br>Enter a financial disclosure statement that describes the sources of funding for the work included in this submission. Review the submission guidelines for detailed requirements. View published research articles from *PLOS ONE* for specific examples.<br><br>This statement is required for submission and **will appear in the published article** if the submission is accepted. Please make sure it is accurate.	Y.S. received the Japan Society for the Promotion of Science KAKENHI Grant Number 20K19928. A.H. received the Japan Society for the Promotion of Science KAKENHI Grant Number 20K10467.<br>The funders had no role in study design, data collection and analysis, decision to publish, or preparation of the manuscript.

Powered by Editorial Manager® and ProduXion Manager® from Aries Systems Corporation

**Unfunded studies**<br>Enter: *The author(s) received no specific funding for this work.*<br><br>**Funded studies**<br>Enter a statement with the following details:<br>• Initials of the authors who received each award<br>• Grant numbers awarded to each author<br>• The full name of each funder<br>• URL of each funder website<br>• Did the sponsors or funders play any role in the study design, data collection and analysis, decision to publish, or preparation of the manuscript?<br>• **NO** - Include this sentence at the end of your statement: *The funders had no role in study design, data collection and analysis, decision to publish, or preparation of the manuscript.*<br>• **YES** - Specify the role(s) played.<br><br>* typeset	
**Competing Interests**<br><br>Use the instructions below to enter a competing interest statement for this submission. On behalf of all authors, disclose any competing interests that could be perceived to bias this work—acknowledging all financial support and any other relevant financial or non-financial competing interests.<br><br>This statement is required for submission and **will appear in the published article** if the submission is accepted. Please make sure it is accurate and that any funding sources listed in your Funding Information later in the submission form are also declared in your Financial Disclosure statement.<br><br>View published research articles from *PLOS ONE* for specific examples.	The authors have declared that no competing interests exist.

**NO authors have competing interests**<br><br>Enter: *The authors have declared that no competing interests exist.*<br><br>**Authors with competing interests**<br><br>Enter competing interest details beginning with this statement:<br><br>*I have read the journal's policy and the authors of this manuscript have the following competing interests: [insert competing interests here]*<br><br>* typeset	
**Ethics Statement**<br><br>Enter an ethics statement for this submission. This statement is required if the study involved:<br><br>• Human participants<br>• Human specimens or tissue<br>• Vertebrate animals or cephalopods<br>• Vertebrate embryos or tissues<br>• Field research<br><br>Write "N/A" if the submission does not require an ethics statement.<br><br>General guidance is provided below. Consult the submission guidelines for detailed instructions. **Make sure that all information entered here is included in the Methods section of the manuscript.**	N/A

Powered by Editorial Manager® and ProduXion Manager® from Aries Systems Corporation

**Format for specific study types**<br><br>**Human Subject Research (involving human participants and/or tissue)**<br>• Give the name of the institutional review board or ethics committee that approved the study<br>• Include the approval number and/or a statement indicating approval of this research<br>• Indicate the form of consent obtained (written/oral) or the reason that consent was not obtained (e.g. the data were analyzed anonymously)<br><br>**Animal Research (involving vertebrate animals, embryos or tissues)**<br>• Provide the name of the Institutional Animal Care and Use Committee (IACUC) or other relevant ethics board that reviewed the study protocol, and indicate whether they approved this research or granted a formal waiver of ethical approval<br>• Include an approval number if one was obtained<br>• If the study involved *non-human primates*, add *additional details* about animal welfare and steps taken to ameliorate suffering<br>• If anesthesia, euthanasia, or any kind of animal sacrifice is part of the study, include briefly which substances and/or methods were applied<br><br>**Field Research**<br><br>Include the following details if this study involves the collection of plant, animal, or other materials from a natural setting:<br>• Field permit number<br>• Name of the institution or relevant body that granted permission	
**Data Availability**<br><br>Authors are required to make all data underlying the findings described fully available, without restriction, and from the time of publication. PLOS allows rare exceptions to address legal and ethical concerns. See the PLOS Data Policy and FAQ for detailed information.	No - some restrictions will apply

A Data Availability Statement describing where the data can be found is required at submission. Your answers to this question constitute the Data Availability Statement and **will be published in the article**, if accepted.<br><br>**Important:** Stating 'data available on request from the author' is not sufficient. If your data are only available upon request, select 'No' for the first question and explain your exceptional situation in the text box.<br><br>Do the authors confirm that all data underlying the findings described in their manuscript are fully available without restriction?	
**Describe where the data may be found in full sentences. If you are copying our sample text, replace any instances of XXX with the appropriate details.**<br><br>• If the data are **held or will be held in a public repository**, include URLs, accession numbers or DOIs. If this information will only be available after acceptance, indicate this by ticking the box below. For example: *All XXX files are available from the XXX database (accession number(s) XXX, XXX.).*<br>• If the data are all contained **within the manuscript and/or Supporting Information files**, enter the following: *All relevant data are within the manuscript and its Supporting Information files.*<br>• If neither of these applies but you are able to provide **details of access elsewhere**, with or without limitations, please do so. For example:<br><br>*Data cannot be shared publicly because of [XXX]. Data are available from the XXX Institutional Data Access / Ethics Committee (contact via XXX) for researchers who meet the criteria for access to confidential data.*<br><br>*The data underlying the results presented in the study are available from (include the name of the third party*	Due to Twitter's terms of service, we cannot share the raw data used in this study. However, we share the daily/weekly number of tweets, the number of tweets per account, and frequently appeared words in the supplement for replication purposes.

and contact information or URL).<br>• This text is appropriate if the data are owned by a third party and authors do not have permission to share the data.<br><br>* typeset	
Additional data availability information:	

Manuscript Click here to access/download;Manuscript;RubellaSNS_v0.pdf

Long-term observation of social media posts about rubella in Japan

Yukie Sano[1], Ai Hori[2],

1 Faculty of Engineering, Information and Systems, University of Tsukuba, Tsukuba, Ibaraki, Japan
2 Faculty of Medicine, University of Tsukuba, Tsukuba, Ibaraki, Japan

*sano@sk.tsukuba.ac.jp

Abstract

Although rubella is an infectious disease that can be prevented by vaccination, there have been periodic epidemics in Japan, mainly among adult males. One of the reasons for this is the lack of interest in vaccination among the target adult male population. To empirically observe interest in rubella, we collected and analyzed Twitter posts about rubella in Japanese between January 2010 and May 2022. We found that the weekly number of rubella reports and the number of Twitter posts fluctuate simultaneously, with an increase in both during the 2012-2013 rubella epidemic. Furthermore, during the 2018 rubella epidemic, the number of tweets increased due to the start of the rubella routine vaccination program and the use of cartoons to raise awareness. While 80% of the accounts posted three times or fewer during the period, some accounts posted multiple times per day for more than 12 years. Most of the content posted was medical information, on topics such as vaccines and antibodies.

Introduction

Rubella is a vaccine-preventable disease; however, periodic outbreaks have occurred in Japan, mainly among adult men who did not have the opportunity to be vaccinated during their childhood. In the 2012-2013 rubella epidemic, there were more than 14,000 reported cases of infection [1], and 45 cases of congenital rubella syndrome were reported [2]. After that, the Japanese government set a goal of eliminating rubella by 2020 [3]. As specific measures against rubella, the Japanese government recommended that women who planned to conceive and their families be checked for vaccination history. The municipal government offered partial expense support for rubella antibody testing and vaccination [4]. However, the voluntary rubella vaccination rate remained low, especially among men who did not plan to have babies [5]. A catch-up vaccination program for adult men was not developed at that time.

Then the rubella outbreak occurred again in 2018. The U.S. Centers for Disease Control and Prevention (CDC) recommended that unvaccinated people and pregnant women refrain from traveling to Japan [6]. Due to concerns about the negative impact of the rubella outbreak on the upcoming 2020 Rugby World Cup and Tokyo Olympic/Paralympic Games [7], the rubella routine vaccination program was initiated in 2019. In the new rubella routine vaccination program, free vaccination was offered to men born between fiscal years 1962 and 1978 who were preliminarily tested for antibodies freely and found to have low antibody titer [8]. The promotion measures

were introduced to allow working-age men to take rubella antibody tests on the occasion of annual health checkups at their workplaces or in their communities [9]. However, use of the catch-up program remained low at 20% [10] of the eligible population at the end of 2021, and there is a risk of a resurgence of rubella outbreaks under the current circumstances. Therefore, the Japanese government decided to extend the rubella routine vaccination program for three years from 2022 [11], and further promotion of the program is required.

One of the reasons for the stagnation of routine rubella vaccination is the lack of interest among the target population, men in their 40s and 50s. Compared to women, adult men are known to be less likely to take actions such as checking rubella vaccination history, antibody testing, and immunization [12], and tend to be less likely to perceive it as a problem related to themselves. Factors associated with the use of the routine immunization program include knowing of the government's recommendation, having acquaintances who have used the program, and being aware that their generation did not have the opportunity to be immunized [13]. In order to arouse widespread interest in rubella, the Ministry of Health, Labor and Welfare (MHLW) in Japan has begun actively using Twitter, one of the popular social networking services (SNS) in Japan, to announce rubella-related information. Similarly, rubella sufferers and doctors are also actively using Twitter to disseminate information. According to a 2021 survey, the percentage of individuals using SNS in Japan reached 78.7% [14]. Those in their 30s and 40s, with usage rates of 89.5% and 87.3% respectively, are the generation that uses SNS the most. The number of individuals using SNS has been increasing in almost all age groups, from teens to 80s; the growth has been particularly significant in the 60- and 70-year-old age groups.

Monitoring of infectious diseases using SNS such as Facebook and Twitter has been active in public health research targeting measles [15–20], human papillomavirus (HPV) [21–23], and zika [24] even before the Covid-19. During the rubella epidemic in the Netherlands in 2013, the number of rubella cases and online news increased simultaneously, leading to an increase in tweets (Twitter posts) [15]. It is also reported that tweets increase in response to a main political event such as mandatory immunizations in Italy [20]. Public agencies such as the CDC and World Health Organization (WHO) were influential in disseminating information during the zika outbreak in 2015-2016 [24]. These previous studies have mainly analyzed a couple of years of epidemics.

On the other hand, few studies exist that track the topic of a specific infectious disease, especially rubella, over ten years or more, including periods of no epidemic. Here, we have analyzed long-term posts on SNS for rubella, which has been a recurring epidemic in Japan. In particular, we have analyzed posts on Twitter, which is said to have 45 million users in Japan as of 2017 [25]. Note that there has been little debate in favor of or against the rubella vaccine in Japan, but the lack of interest in rubella has been problematic. Therefore, it is crucial to observe how this pubic interest in rubella has evolved. On the other hand, it is difficult to empirically capture public interest, which is often vaguely defined. As a means of overcoming this difficulty, we empirically clarify it by observing the posts on the Twitter space, which engages a considerable population in Japan. For example, it is important to understand the basic information about a number of rubella-related posts and the impact of the MHLW's announcements on rubella to promote the rubella vaccination in Japan. Furthermore, tracking rubella-related tweets over such a long period is important from an academic perspective. This is because little is known about how the topic of infectious disease is handled during the period when an epidemic is not occurring.

In the following, we first provide an overview of the data and the main analytical methods used. Next, we present the results of our analysis of long-term fluctuations and

the content of the posts. Finally, we summarize the results of this analysis and discuss the significance of the study.

Materials and methods

Data

In this study, Twitter Academic API was used to retrieve tweets about rubella written in Japanese. The acquisition condition was defined as tweets containing "rubella" expressed in either Kanji (Chinese characters) or Hiragana from January 1, 2010, to May 31, 2022. We collected these rubella-related tweets posted publicly on Twitter.

Usually, when text-based tweets are collected, they may contain spam tweets intended as advertisements. In the case of rubella in Japanese, there were almost no spam-related tweets, probably because "rubella" is not a very common word. Therefore, no special filtering process was used in this data collection.

Overall, 2,410,868 tweets from 575,311 accounts were collected in total. We separated an original tweet and a retweet based on whether a tweet included "RT" at the beginning of the body of the tweet. Therefore, quoted retweets that precede the quoted text with their own opinion were treated as original tweets. The percentage of retweets was 64.9%. The average number of tweets (sum of both original tweets and retweets) per day for the entire period covered was 532 (187 original tweets and 345 retweets). Thus, daily rubella-related tweets were very small compared to the Japanese Twitter population.

Overview of analysis

This study aimed to provide a basic analysis of rubella-related Twitter posts; therefore, we focus on the following three points.

- Time series of rubella-related tweets and rubella reports
- Number of tweets per account
- Visualization of content

Time series of tweets and reports Weekly counts of tweets were conducted to determine the overall fluctuation of tweets about rubella and to compare them with the number of rubella reports [1]. The number of rubella reports was counted by downloading the information available on the website (`https://www.niid.go.jp/niid/ja/hassei/3086-rubella-sokuhou-rireki.html`). To be consistent with the time period for the reported cases, we used the tweet time period of ten years from 2012.

Number of tweets per account To determine a typical account that posts about rubella, we counted the number of tweets per account throughout the entire period from 2010 to 2022. Some accounts were individually web-surveyed to determine whom they were inferred to be based on publicly available information.

Visualization of content In order to understand tweet content, we visualized tweets using wordcloud (`http://amueller.github.io/word_cloud/`). Here, as a pre-processing of the tweet body, the words were segmented using NEologd [26], a dictionary that also supports Internet slang, omitting punctuation and symbols.

Results

Time series of tweets and reports

Figure 1 shows the weekly number of rubella reports and the rubella-related tweets. The changes in original tweets and retweets were almost synchronous, with a correlation coefficient $r = 0.712$ ($p < .001$). The overall changes was also similar to the number of rubella reports, with an increase in the number of tweets in 2013 and 2018. In particular, the number of reports and the number of original tweets fluctuate simultaneously, with a correlation coefficient $r = 0.694$ ($p < .001$). On the other hand, the correlation coefficient between the number of reports and the number of retweets was less similar, $r = 0.210$ ($p < .001$).

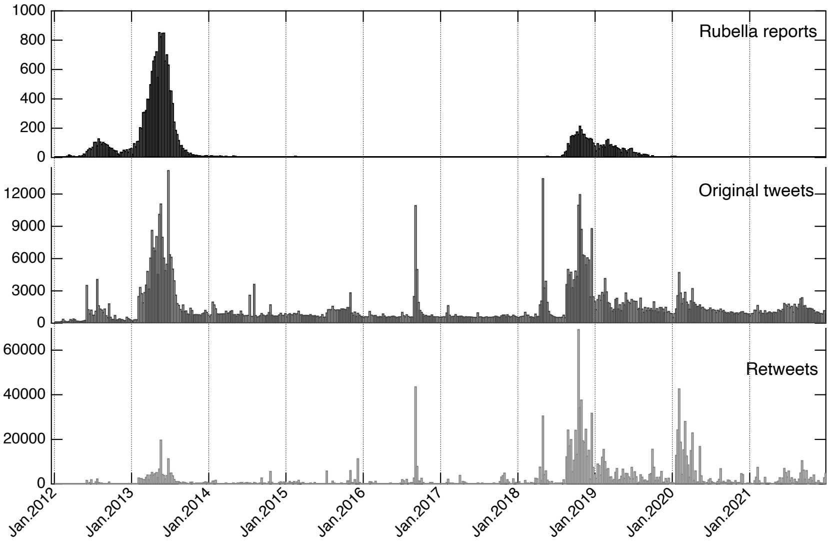

Fig 1. Weekly time series of tweets and rubella reports. From the top row, the weekly number of rubella reports, original tweets, and retweets, respectively.

On a daily basis, the day with the largest number of original tweets was December 11, 2018 with 4,820 tweets. This was the day the MHLW announced that vaccinations would be free for three years for men aged 39 to 56 as a measure against rubella. Note that Fig. 1 shows weekly numbers that have the highest peak in the middle of 2018 for original tweets.

The day with the most retweets was October 11, 2018, with 27,068 retweets. This was the day that the episode about rubella in the cartoon “Konodori” was released for free. “Konodori” is a popular medical cartoon featuring an obstetrician. On the day of the event, the cartoon created many tweets because it was published online for free to stimulate interest in rubella.

Interestingly, the median number of daily tweets increased more than double before and after 2018, when the second epidemic occurred; the value varied from 153 (95 original tweets and 57 retweets) in 2017 to 555 (219 original tweets and 333 retweets) in 2019.

Number of tweets per account

~~We checked how many posts (both original tweets and retweets) about rubella were made per account during twelve years and five months~~. The results showed that

approximately 320 thousand accounts, or 56.6% of the total accounts, made only one tweet. The next most common was 90 thousand accounts with two tweets, followed by 40 thousand accounts with three tweets (Fig. 2(a)).

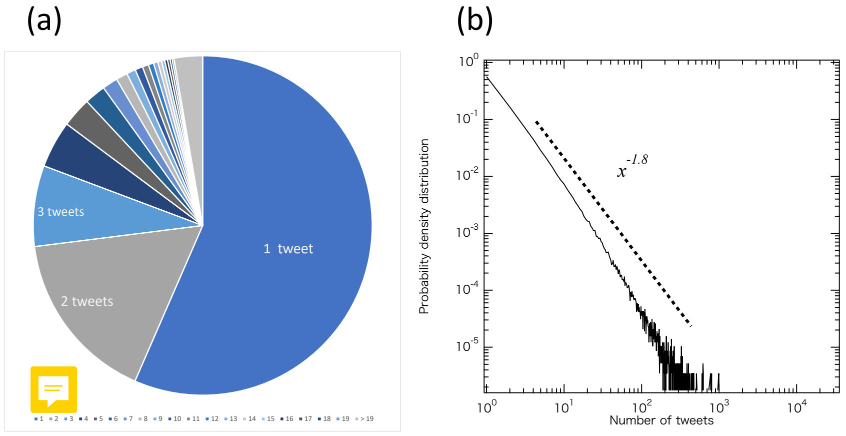

Fig 2. Number of tweets per account. (a) Ratio of tweets per account. The majority of the tweets were made by only a small number of users. (b) Distribution of number of tweets per account (logarithmic on both axes). The typical power distribution with an exponent of 1.8.

On the other hand, one account made 33,073 posts during this period. This account seemed to be a doctor with about ten thousand followers, and it has been regularly tweeting about rubella, using a bot that automatically posts tweets. The next highest account seems to be a family member of those affected by congenital rubella syndrome who made 19,565 tweets. This account was very active in the rubella eradication effort, and although it does not seem to use any automated framework, it made numerous posts. In addition, the official account of the MHLW (@MHLWitter) was also active in tweeting information on rubella, posting 1,136 tweets during the period. MHLW was the 56th most popular among the 575,311 accounts. Thus, overall, the number of tweets about rubella per account was highly skewed and showed power-law distribution $P(x) \sim x^{-\alpha}$ with its exponent α estimated [27] as 1.8 (Fig. 2(b)).

Visualization of written content

Figure 3 shows wordclouds created with the top 150 most frequent words in original tweets and retweets, respectively. The top 10 frequent words are shown in Table 1. The top 200 most frequent words with original Japanese forms appear in S1 Appendix.

As an overall trend, many of the words that frequently appeared in both original tweets and retweets were common to the rubella-related tweets. Those common words were medical terms such as "vaccination," "vaccine," and "antibody," suggesting that information about rubella is correctly conveyed within the Twitter space.

Figure 4 shows wordclouds of original tweets on December 10, 2018 (the day with the most original tweets) and retweets on October 11, 2018 (the day with the most retweets). The top 10 frequent words are shown in Table 2.

The original tweet was on December 11, 2018, when the MHLW announced that antibody testing and vaccination would be free for men aged 39 to 56. At the time, major newspapers reported the news, and many people shared it on the web. The highest number of retweets occurred on October 11, 2018, with several characteristic

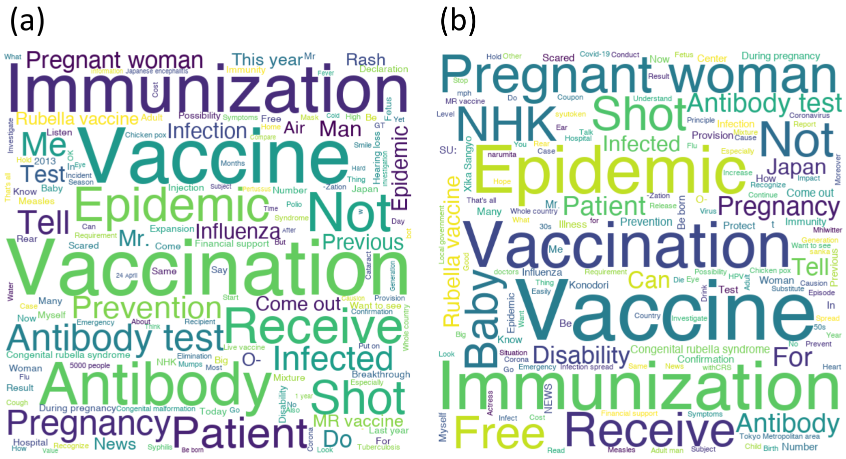

Fig 3. Wordcloud created with the top 150 most frequently occurring words during the entire period. (a): Original tweets only. (b): Retweets only.

Table 1. Top 10 most frequent words of whole period.

	**Original tweets**		**Retweets**	
Rank	Word	Frequency	Word	Frequency
1	Vaccination	196243	Vaccine	761784
2	Vaccine	195454	Immunization	452897
3	Measles	168357	Epidemic	431119
4	Immunization	158337	Vaccination	398034
5	Antibody	120058	Infection	377218
6	Infection	104715	Measles	370225
7	Not	99549	Pregnant woman	301967
8	Receive	85224	Man	263005
9	Epidemic	76446	Not	258418
10	Shot	73710	NHK	253100

Table 2. Top 10 most frequent words of the specific day.

	**Original tweets** (Dec. 12th, 2018)		**Retweets** (Oct. 11th, 2018)	
Rank	Word	Frequency	Word	Frequency
1	Vaccination	3009	Release	26394
2	Man	2599	Konodori	25104
3	Free	2537	Free	24357
4	Antibody	1380	Episode	23568
5	Test	1101	Epidemic	19805
6	56 years old	990	Receive	15019
7	Not	897	Tokyo Metropolitan area	14782
8	Immunization	889	Center	14232
9	39	856	Adult man	14054
10	Receive	721	Pregnant woman	13888

words ("release," "Konodori," "free") appearing that were not present during the other periods. The event caused the episode about rubella in the cartoon "Konodori" was released for free to arouse public interest in rubella. It is suggested that press releases

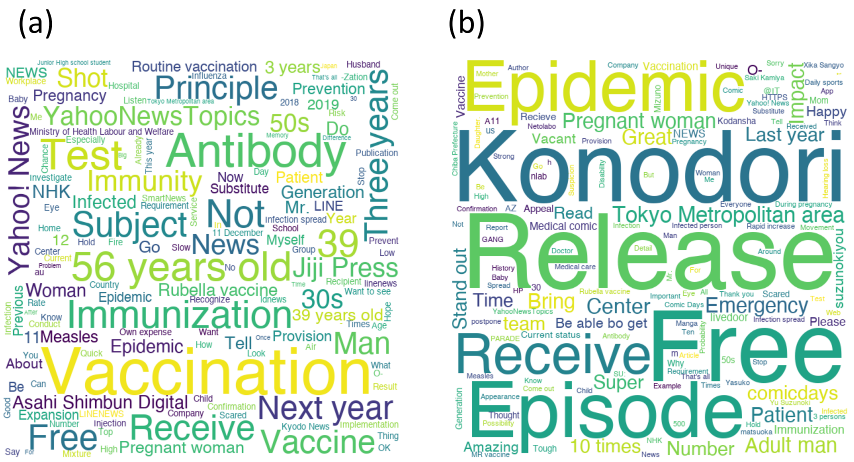

Fig 4. Wordcloud created with the top 150 most frequently occurring words at the specific day. (a): Original tweets only (Dec. 12th, 2018). (b): Retweets only (Oct. 11th, 2018).

using tie-up projects with popular content can be effective in promoting the spread of public health information.

The word "free" was shared on both days, with the largest number of original tweets and retweets, even though it appeared in separate contexts. These were free vaccinations in the case of the original tweet and free cartoon publication in the case of the retweet. The word "free" may be attractive and have attracted people, and it is important to examine this finding for future health-promoting campaigns.

Summary and Discussion

We presented a chronological change in the Japanese Twitter space regarding rubella for more than ten years. The number of weekly rubella reports and tweets showed similar fluctuations, especially for original tweets. Even rubella-related tweets are weak signals; we found that they have increased in recent years, particularly in response to news and related events. These results are consistent with SNS analyses of measles and other diseases conducted in other countries [15, 18, 20].

A small number of accounts continue to disseminate information on rubella actively for more than ten years. Although the Japanese MHLW was in the top 1% of all rubella-related accounts, it was hardly the most influential in the Japanese Twitter space for rubella. Instead, individuals such as a doctor and a family member with congenital rubella syndrome were most active. This was somewhat different compared to the results in the U.S., where the WHO and CDC were said to be influential [24]. Rubella-related information dissemination in Japanese Twitter space can be considered to be supported by this kind of grassroots movement. The majority of accounts that posted about rubella tweeted only once or twice. This suggests that the majority of accounts are interested in something other than rubella on a daily basis. These results suggest that Twitter's activities to raise awareness using tie-up projects about rubella may reach people who were not interested in the disease in some sense.

Medical terms such as vaccination and antibodies were frequently used in tweets about rubella. There were also many tweets containing "pregnant women." This suggests that the majority of the tweets conveyed correct medical information that

pregnant women infected with rubella may have children with congenital rubella syndrome, which can be prevented by vaccination. We did not find anti-vaccine comments by checking rubella-related tweets randomly. This is very different from the vaccine discussion of other diseases [19, 21, 28]. Nevertheless, the current SNS information explosion, especially regarding health, known as the "infodemic," [29–31] should not be underestimated for the future situation of rubella.

Here we analyzed Japanese tweets about rubella over a long period of time. In Japanese Twitter space, interest in rubella has never been high, and the number of tweets is tiny compared to that of Covid-19 tweets. On the other hand, as rubella is a vaccine-preventable infectious disease, it is possible to eliminate it from Japan if the public is appropriately aware of the disease and vaccinated against it. Therefore, empirically observing and recording not only the number of rubella cases, but also the social interest in the disease is highly desirable as a resource for future public health lessons.

Supporting information

S1 Appendix Raw numbers and words used in the study. In the file, we provide raw numbers of tweets and rubella reports in Japan. Also, the file includes that frequently appear words both in Japanese and English.

References

1. National Institute of Infectious Diseases. Epidemiological information on rubella (in Japanese); 2022. `https://www.niid.go.jp/niid/images/epi/rubella/2022/rubella220518.pdf`.

2. Kanai M, Sunagawa T, Kamiya H, Okuno H, Taya K, Oishi K, et al. Report on the results of a follow-up study of 45 cases of congenital rubella syndrome born between 2012 and 2014 (in Japanese). National Institute of Infectious Diseases, Japan; 2018. 3.

3. Ministry of Health L, Welfare. Guidelines for Prevention of Specific Infectious Diseases Related to Rubella (in Japanese); 2014. `https://www.mhlw.go.jp/file/06-Seisakujouhou-10900000-Kenkoukyoku/0000186690.pdf`.

4. Director of Tuberculosis Infectious Disease Division MoHL Health Bureau, Welfare. Notification: Further Thoroughness of Measures to Prevent Outbreaks of Congenital Rubella Syndrome (in Japanese). National Institute of Infectious Diseases, Japan; 2013.

5. Hori A, Wada K, Smith DR. A Socio-Demographic Examination of Adults Responding to Governmental Vaccination Recommendations during the Japanese Rubella Outbreak of 2013. PLoS ONE. 2015;10(6):e0129900.

6. U.S. CDC warns pregnant women against traveling to Japan amid rubella outbreak; 2018. `https://www.japantimes.co.jp/news/2018/10/24/national/science-health/u-s-cdc-warns-pregnant-women-traveling-japan-amid-rubella-outbreak/`.

7. Kato H. The Fifth Routine Immunization Phase So Far and Future after the 2018 Rubella Outbreak (in Japanese). National Institute of Infectious Diseases, Japan;.

8. Ministry of Health L, Welfare. Additional Measures for Rubella (in Japanese); 2022. https://www.mhlw.go.jp/stf/seisakunitsuite/bunya/kenkou_iryou/kenkou/kekkaku-kansenshou/rubella/index_00001.html.

9. Ministry of Health L, Welfare. Measures to improve the implementation rate of rubella countermeasures (antibody testing) (in Japanese); 2020. https://www.mhlw.go.jp/content/10906000/000590968.pdf.

10. Ministry of Health L, Welfare. Current status of additional measures regarding rubella (in Japanese); 2021. https://www.mhlw.go.jp/content/10906000/000868298.pdf.

11. Ministry of Health L, Welfare. Minutes of the 46th Meeting of the Basic Immunization Policy Subcommittee of the Subcommittee on Immunization and Vaccine of the Health Sciences Council and the 57th Meeting of the Subcommittee on Infectious Diseases of the Health Sciences Council (joint meeting) (in Japanese); 2021. https://www.mhlw.go.jp/stf/newpage_23317.html.

12. Norizuki M, Hori A, Wada K. Factors associated with adults' actions to confirm their own rubella immune status in Japan's drive toward rubella elimination: Cross-sectional online survey of non-healthcare workers in their 20s to 40s. Environ Health Prev Med. 2021;26(1):77.

13. Hori A, Yoshii S, Isaka Y, Wada K. Factors associated with participation in an ongoing national catch-up campaign against rubella: a cross-sectional internet survey among 1680 adult men in Japan. BMC Public Health. 2021;21(1):292.

14. Ministry of International Affairs and Communications. 2021 White Paper on Information and Communications in Japan; 2022.

15. Mollema L, Harmsen IA, Broekhuizen E, Clijnk R, De Melker H, Paulussen T, et al. Disease detection or public opinion reflection? Content analysis of tweets, other social media, and online newspapers during the measles outbreak in The Netherlands in 2013. J Med Internet Res. 2015;17(5):e128.

16. Broniatowski DA, Hilyard KM, Dredze M. Effective vaccine communication during the disneyland measles outbreak. Vaccine. 2016;34(28):3225–3228.

17. Radzikowski J, Stefanidis A, Jacobsen KH, Croitoru A, Crooks A, Delamater PL. The Measles Vaccination Narrative in Twitter: A Quantitative Analysis. JMIR Public Health Surveill. 2016;2(1):e1.

18. Gunaratne K, Coomes EA, Haghbayan H. Temporal trends in anti-vaccine discourse on Twitter. Vaccine. 2019;37(35):4867–4871.

19. Cossard A, Morales GDF, Kalimeri K, Mejova Y, Paolotti D, Starnini M. Falling into the echo chamber: the Italian vaccination debate on Twitter. In: Proceedings of the International AAAI conference on web and social media. vol. 14; 2020. p. 130–140.

20. Ajovalasit S, Dorgali VM, Mazza A, d'Onofrio A, Manfredi P. Evidence of disorientation towards immunization on online social media after contrasting political communication on vaccines. Results from an analysis of Twitter data in Italy. PLoS ONE. 2021;16(7):e0253569.

21. Dunn AG, Leask J, Zhou X, Mandl KD, Coiera E. Associations Between Exposure to and Expression of Negative Opinions About Human Papillomavirus Vaccines on Social Media: An Observational Study. J Med Internet Res. 2015;17(6):e144.
22. Surian D, Nguyen DQ, Kennedy G, Johnson M, Coiera E, Dunn AG. Characterizing Twitter Discussions About HPV Vaccines Using Topic Modeling and Community Detection. J Med Internet Res. 2016;18(8):e232.
23. Lama Y, Chen T, Dredze M, Jamison A, Quinn SC, Broniatowski DA. Discordance Between Human Papillomavirus Twitter Images and Disparities in Human Papillomavirus Risk and Disease in the United States: Mixed-Methods Analysis. J Med Internet Res. 2018;20(9):e10244.
24. Stefanidis A, Vraga E, Lamprianidis G, Radzikowski J, Delamater PL, Jacobsen KH, et al. Zika in Twitter: Temporal Variations of Locations, Actors, and Concepts. JMIR Public Health Surveill. 2017;3(2):e22.
25. Twitter Japan. The number of monthly users in Japan has exceeded 45 million. (in Japanese); 2017. `https://twitter.com/TwitterJP/status/923671036758958080`.
26. Toshinori Sato TH, Okumura M. Implementation of a word segmentation dictionary called mecab-ipadic-NEologd and study on how to use it effectively for information retrieval (in Japanese). In: Proceedings of the Twenty-three Annual Meeting of the Association for Natural Language Processing. The Association for Natural Language Processing; 2017. p. NLP2017–B6–1.
27. Alstott J, Bullmore E, Plenz D. Powerlaw: A python package for analysis of heavy-tailed distributions. PLoS ONE. 2014;9(1).
28. Miyazaki K, Uchiba T, Tanaka K, Sasahara K. Characterizing the Anti-Vaxxers' Reply Behavior on Social Media. In: IEEE/WIC/ACM International Conference on Web Intelligence and Intelligent Agent Technology. WI-IAT '21. Association for Computing Machinery; 2021. p. 83–89.
29. Zarocostas J. How to fight an infodemic. Lancet. 2020;395(10225):676.
30. The Lancet Infectious Diseases. The COVID-19 infodemic. Lancet Infect Dis. 2020;20(8):875.
31. Saeki S, Shimato M. Combatting infodemics for a sustainable healthcare system. Nihon Koshu Eisei Zasshi. 2021;68(6):444–445.

Click here to access/download
Supporting Information
Plos_data.xlsx
