# Supplementary material for: 12-year observation of tweets about rubella in Japan: A retrospective infodemiology study
Source: PLoS One. 2023 May 8;18(5):e0285101. doi: 10.1371/journal.pone.0285101 (PMC10166556; doi:10.1371/journal.pone.0285101)
Supplement: Attachment — Submitted filename: Responses_v1.pdf [file pone.0285101.s003.pdf]

Black fonts: Original comments
Red fonts: Our responses
Blue fonts: Revisions in the manuscript

**Additional Editor Comments:**
Title:
1. The term "Long-term" in the title is misleading. Please present number of years
2. The term "social media posts" in the title is misleading. Nowadays, hundreds of social media platforms are available. You only assessed Twitter. Please correct.
3. Add type of study in the title. For instance, A Retrospect Infodemiology Study
-> Thank you for the comment for our title. We reconsider our title as "12-year observation of tweets about rubella in Japan: a retrospective infodemiology study".

Abstract:
1. Expand the content related to methods
-> We added content related to our methods in our abstract.
We examined time series, number of tweets per account, tweeted contents, and retweet network.

Introduction:
1. Clearly state the aim or hypothesis of the study in the abstract and the last paragraph of the introduction.
-> We clarify our aim of the research as follows.
To clarify the reality of the discussion about rubella, and to provide basic resource for enlightening activities for rubella prevention, (abstract)

Purpose of the study
The purpose of this study is to clarify the reality of the discussion about rubella on Japanese Twitter space, and to provide basic data for enlightening activities on rubella prevention. (the last subsection in Introduction)

Materials and methods:
1. Add method of correlation assessment and the software used for statistical analysis.
-> We added the method of the correlation assessment.
We then operated `StatsLinearCorrelationTest` by Igor Pro 9.0 (WaveMetrics, Inc.) to calculated Pearson's correlation coefficient. (Methodology > Overview of analysis > Time series of tweets and reports)

2. Do not present results in the methods section. For instance, "Overall, 2,410,868 tweets from 575,311 accounts were collected in total." and "The percentage of retweets was 64.9%. The average number of tweets (sum of both original tweets and retweets) per day for the entire period covered was 532 (187 original tweets and 345 retweets)."
-> Thank you for the important points. We moved above sentences to the beginning of the Result section.

3. Addition of Twitter Network Analysis will help readers to know about active Twitter accounts related to rubella and the network of relationship between them. The

following articles will help you in this regard. doi: 10.1177/0165551515608733 and doi:10.1017/dmp.2020.347

-> Thanks for the meaningful suggestion. We newly added the Twitter retweet network analysis in our manuscript.

4. Addition of Twitter Sentiment Analysis will increase quality of your study.

-> Thanks for the meaningful suggestion. This time we did not include sentiment analysis, but we will consider is as our future work:

In the future, a more detailed analysis through topic analysis [44] and sentiment analysis [45] will also be necessary for the deeper understanding of rubella context.

(last paragraph in Discussion)

Results:

1. Add 95% confidence interval and p value for correlations

-> We added 95% confidence interval in Results.

$r = 0.71$ ($p < .001$, 95%CI 0.67–0.75)

$r = 0.69$ ($p < .001$, 95%CI 0.65–0.74)

$r = 0.21$ ($p < .001$, 95%CI 0.13–0.29)

2. Do not duplicate presentation of the frequent words in wordcloud and table. Please delete tables.

-> We deleted the tables from the manuscript.

3. Present top twitter accounts. Are they related to Members of the public, Practitioners (doctors, other healthcare professionals) or Scientists?

-> Thank you for the suggestions. We clarity the top Twitter accounts and even stated the screen names in case we had their permission.

Discussion:

1. Compare your results related to correlation coefficient and other findings with similar studiers which assessed relationship between number of tweets and disease incidence.

-> Thank you for the insightful comments. We added the following sentences in Discussion.

Similar correlations between tweets and disease surveillance are reported in other studies. For instance, in the US, a study on influenza revealed a strong correlation (r = 0.93) between the number of tweets classified as valid and the rates of influenza-like illness, although the strength of the relationship varied across different cities [40]. In the case of the 2013 measles outbreak in the Netherlands, the correlation between tweets and disease incidence was reported to be r = 0.56 [16]. Not only infectious diseases but also hay fever has been reported correlation (r = 0.97) in the UK [41]. In Japan's case, tweets related to allergic rhinitis were highly correlated (r = 0.84) with their drug sales [42]. We reached similar results using longer-term data.

2. Do not use ambiguous terms such as "a doctor and a family member". Clearly present the Twitter account name.

-> We clarity the top Twitter accounts and mention their screen names in case we had their permission.

3. Only discuss about the outcomes presented in the result section. Do not present new results such as “We did not find anti-vaccine comments by checking rubella-related tweets randomly.”
4. Add the limitation of this study.
5. Provide some suggestions for future researches.

-> We appreciate your very important comments. We revised the overall structure of Results and Discussion sections. In the final paragraph of Discussion, we added the limitation and future suggestions of this study.

**Reviewer #1:**

Major comments:

・Introduction section is too long. The authors should summarize the key points.

-> Thank you for the suggestion. To make the Introduction section easier to read, we have divided it into subsections. In addition, redundant text has been removed.

・Based on the results, it seems that the authors conduct the statistical analysis. However, there are no information of statistical analysis in the method section

-> We added the method of the correlation assessment.

We then operated `StatsLinearCorrelationTest` by Igor Pro 9.0 (WaveMetrics, Inc.) to calculated Pearson’s correlation coefficient. (Methodology > Overview of analysis > Time series of tweets and reports)

・The authors should add the limitation of this study.

・Discussion section is too short. The authors should add the key points, especially in implication using the results of this study.

-> Thank you for the important comments. We received similar comments from the editor and the reviewers to our Discussion. Therefore, we revised the overall structure of Discussion section. In the final subsection of the Discussion section, we added the limitation and future suggestions of this study.

Minor comments:

・Please refer this article about situation of rubella in Japan (PMID: 32330153) in the introduction.

-> Thank you for the reference. We referred PMID: 32330153 in our Introduction as [3].

In the 2012-2013 rubella epidemic, there were more than 14,000 reported cases of infection [1], and 45 cases of congenital rubella syndrome were reported [2] with the key clinical characteristic of conjunctivitis [3].

**Reviewer #2:**

The manuscript entitled "Long-term observation of social media posts about rubella in

Japan" was written well and had a sound methodology. However, some revisions were needed, particularly for the discussion part.
Another point that should be considered was the subject of the manuscript which was a local subject. But should be noted that rubella reminded an important health issue for the Japanese.
I hope you find the review constructive.
Please see the attached file.
-> Thank you for your kind and constructive comments. As the editor and other reviewers pointed, we revised overall structure of our Discussion. We also read your attached file carefully and reflected all of them.

In the attached file: Also, the authors might reach a simplistic conclusion. The existence of a word does not always implicate support. They might combine with negative words.
-> Thanks for bringing this important point to our attention. We added the limitation of this word frequency analysis.
Finally, this study focused on presenting a basic analysis of discussions about rubella in Japanese Twitter space. For example, simple word frequency is not a sufficient measure of context because it is sometimes used with a negative word. (Discussion > Limitation and future study)

**Reviewer #3:**
The paper "Long-term observation of social media posts about rubella in Japan" reviewed and I found it can not be accepted in this form and needs major revisions.
-> We appreciate your careful reviewing of our manuscript. We answer each of your following comment.

- The type of vaccination should be specified.

-> We added information of the type of vaccination in Introduction.
Rubella is a vaccine-preventable disease; rubella-containing vaccine and measles-rubella vaccine are approved in Japan as of 2023. (the beginning of Introduction)

- What is the reason that this disease is more common in adult men?

-> Because Japanese adult men did not have the opportunity to be vaccinated during their childhood. We added following sentences in the beginning of the Introduction.
While women of the same age had the opportunity to be vaccinated, men born before April 1, 1979 did not have the opportunity to be vaccinated even once [1]. (the first paragraph of Introduction)

- Measles is an infectious disease that can be prevented by vaccination, but there have been periodic epidemics in Japan, mainly among adult men.
- Most of the content posted was medical information, on topics such as vaccines and antibodies. What do you mean?
- The English must be checked.

-> We carefully clarified our sentences in our manuscript. This revised manuscript was proofed by a professional firm.

- The discussion part is very inadequate, it should be compared with similar studies in order to understand the superiority of the study.
-> Thank you for the important comments. We received similar comments from the editor and the other reviewers. We revised the overall structure of Discussion section as well as referring the similar studies.

-The references contain some relevant and recent work, but can be increased to strengthen the manuscript. I recommend the authors to consult and cite some relevant form the list. the following papers due to their importance.
-> Thank you for the reference. We referred them and added more papers in the manuscript. As a result, we referred 45 papers in the revised manuscript.

- The structure of this paper needs to be improved.
-> We reconsider overall structure of the manuscript. We segmented the Introduction, moved some part of the Results to Discussion, newly added Conclusions.
